# Quantitative analysis of the intra-beam respiratory motion with baseline drift for respiratory-gating lung stereotactic body radiation therapy

**DOI:** 10.1093/jrr/rrab098

**Published:** 2021-10-29

**Authors:** Kenji Yasue, Hiraku Fuse, Satoshi Oyama, Koichi Hanada, Kazuya Shinoda, Hideaki Ikoma, Tatsuya Fujisaki, Yoshio Tamaki

**Keywords:** residual motion, baseline drift, respiratory-gating method, stereotactic body radiation therapy (SBRT)

## Abstract

This study aimed to quantitatively clarify the baseline drift for each respiratory cycle in two respiratory-gating methods using the intra-beam respiratory motion data of lung cancer patients. The residual motion and dose distribution were calculated based on intra-beam respiratory motion data with the baseline drift. To quantify the baseline drift $\Delta$ during irradiation, it was defined as the inclination between the detected expiration point and the expiration point in the next cycle in the anterior–posterior (AP), cranial–caudal (CC) and left–right (LR) directions obtained using an in-house programme. The baseline drift value reached up to 0.74 mm/s in the CC direction as per the respiratory motion data of 10 patients. The homogeneity index (HI) of the phase-gating method tended to increase because the target was irradiated even when the amplitude position of the target differed from period to period. In contrast, the amplitude-gating method enabled irradiation considering the amplitude position of the target because the gating window was set considering the amplitude position of the respiratory motion. The respiratory-gating methods and respiratory phase in respiratory-gating lung stereotactic body radiation therapy (SBRT) must be determined based on the respiratory motion of the patients.

## INTRODUCTION

Lung stereotactic body radiation therapy (SBRT) has become a viable treatment option for located lung lesions owing to the recent technological advances [1–4]. Baseline drift is caused by differences in the expiration target positions every respiratory cycle and change in the dose distribution, which arise from changes in the target position during irradiation [5–7]. Takao *et al.* reported that baseline drift occurs in about 70% of lung cancer patients who underwent lung SBRT with the real-time tracking radiation therapy (RTRT) system [7]. They examined the baseline drift before and after irradiation. Since the target position moves irregularly during irradiation, it is important to quantify the baseline drift during irradiation.

For respiratory management, body surface signals obtained as the respiratory wave are used in a respiratory-gating device in clinical settings [6, 8–12]. Since the respiratory wave are correlated with the target position, gating irradiation was performed at a specific phase based on the acquired the body surface signal [5, 6]. The first few cycles determine the gating window in device using the body surface signal [6, 8–12]. Therefore, irradiation continues at the set gating window even if baseline drift occurs during the irradiation due to differences in the expiration target positions every respiratory cycle. Hence, irradiation is performed in the same respiratory phase regardless of the baseline drift. An additional problem is that the target position is displaced because of baseline drift in the gating window. The target position displacement is defined as a residual motion and can be evaluated during treatment planning using 4-dimensional computed tomography (CT) [13, 14]. It can also be analysed retrospectively by using a device that can confirm the target during irradiation [13–16].

An RTRT system enables tracking the target position during irradiation. This system comprises four diagnostic X-ray fluoroscopic devices [13–16]. An RTRT system can track the target position using fiducial markers used in gating irradiation. However, the RTRT system is an invasive method for patients as fiducial markers have to be inserted into the body of the patient, and the patients are exposed to X-rays for fluoroscopy. The target position was also adjusted by controlling the couch position before irradiation with each beam.

The respiratory-gating method using a respiratory-gating device is widely used in clinical practice because of the simple gating irradiation process. The phase-gating method generally uses a respiratory-gating device to divide one respiratory cycle into 10 respiratory phases. [9–11]. On the other hand, in the amplitude-gating method, reconstruction is achieved by dividing the amplitude of the respiratory wave into 10 phases [10, 11]. The former method with a reconstruction algorithm implemented by a CT system is used frequently.

This study aimed to quantitatively clarify the baseline drift during irradiation for each respiratory cycle for two respiratory-gating methods using the intra-beam respiratory motion data of lung cancer patients. The residual motion and dose distribution were calculated based on the intra-beam respiratory motion data with the baseline drift to determine the appropriate respiratory-gating method before the respiratory-gating lung SBRT.

## MATERIALS AND METHODS

### Preparing respiratory motion data

#### Patient selection and characterisation

Lung cancer patients treated using an RTRT system (SyncTraX FX4 [Shimadzu, Kyoto, Japan] + TrueBeam STx [Varian Medical Systems, Palo Alto, CA]) at our institution between October 2017 and October 2020 were included in the study. In all patients, three to four fiducial markers were inserted into the bronchi near the target. For treatment, the inserted fiducial marker that was the closest to the target position and correlated with the target motion was used during irradiation. This study was used only the intra-respiratory motion data from these patients treated with the RTRT system to quantify the baseline drift during irradiation. All patients were treated by 3-dimensional conformal radiation therapy (3D-CRT) with the RTRT system. The distance between the centroid of the clinical target volume (CTV) and the fiducial marker was 2–4 cm in all patients. The study was approved by the ethics committee in our institutions.

#### Intra-beam respiratory motion data for lung cancer patients

The respiratory motion data without data loss during treatment were prepared for quantifying the baseline drift during the irradiation. However, the intra-beam respiratory motion data were obtained from the RTRT system because the target position was adjusted by controlling the couch before irradiating with each beam.

**Fig. 1:**
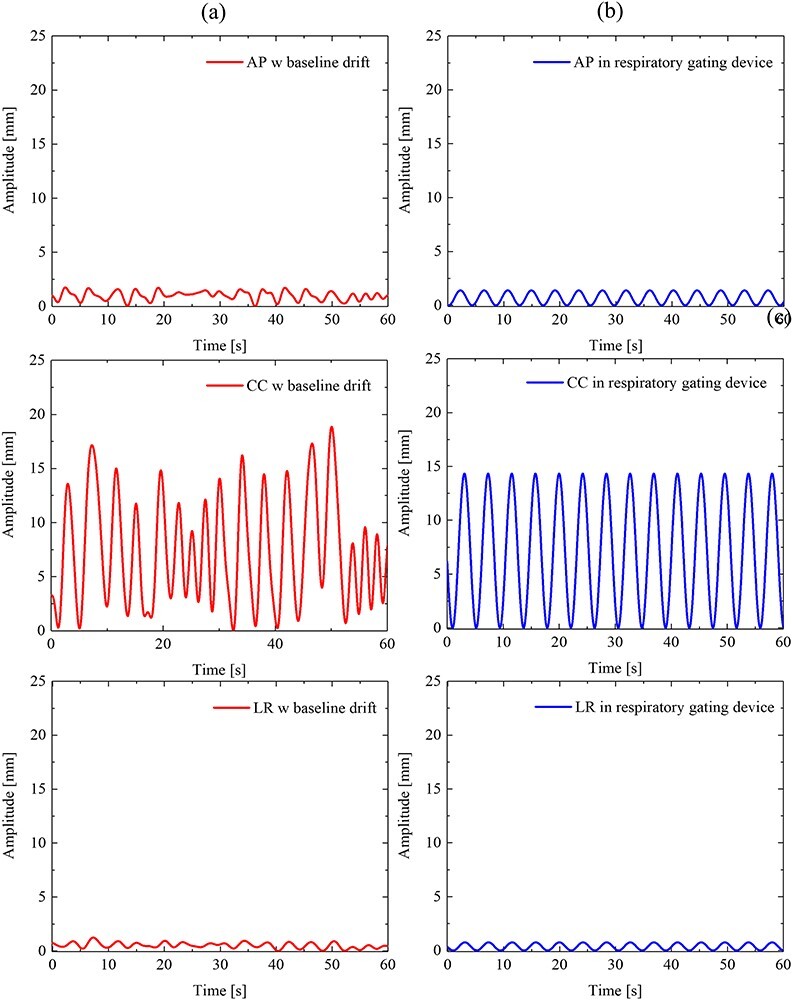
(a) shows the example of the intra-beam respiratory motion data with baseline drift that were obtained from the position of the fiducial markers in patients with lung cancer treated using the RTRT system. Fig. 1(b) shows the respiratory motion data assumed in the respiratory gating device. The gating window was determined in the first few cycles even when the respiratory motion data change in terms of the amplitudes and periods of respiration in each cycle during the irradiation, as shown in Fig. 1(a), Irradiation was performed continuously in the gating window set assuming that the amplitude and period of the patients’ respiration are constant during irradiation, as shown in Fig. 1(b).

In this study, we selected the intra-beam respiratory motion data that were acquired from the position of the fiducial markers in patients with lung cancer without data loss during irradiation. The intra-beam respiratory motion data in the anterior–posterior (AP), cranial–caudal (CC) and left–right (LR) directions were used. An example of the intra-beam respiratory motion data is shown in Fig. 1. The details of the intra-beam respiratory motion data are listed in Table 1. Intra-beam respiratory motion data with no data loss during the irradiation were obtained to form a total of 148 data sets for 10 patients, and these data were analysed. Intra-beam respiratory motion data for each patient covered 6–8 beams per fraction, and there were four fractions, but we excluded the fractions for which data were lost during irradiation to quantify the baseline drift during irradiation. The locations of lung cancer in the intra-beam respiratory motion data of 10 patients were classified into five lung lobes: the right upper lobe (1), right middle lobe (3), left upper lobe (1) and left lower lobe (5). The average of the amplitude and cycle were 10.1 ± 2.2 mm and 3.0 ± 0.6 s, respectively. The average treatment time per beam was 156 s for the 10 patients.

**Table 1:** The details of intra-beam respiratory motion data. We selected intra-beam respiratory motion data that were acquired in the fiducial marker position from patients with lung cancer without data loss during irradiation per beam

Patient No.	Fraction	Beam	Amplitude [mm]	Cycle [s]	Time [s]	Location	Data set
#1	4	6	14.0 ± 2.5	2.7 ± 0.4	166 ± 16	Rt-mid	10
#2	4	6	8.7 ± 1.6	2.3 ± 0.2	179 ± 22	Rt-mid	15
#3	4	6	8.3 ± 0.9	2.1 ± 0.2	166 ± 16	Rt-mid	13
#4	4	7	6.4 ± 1.2	4.7 ± 0.7	166 ± 20	Lt-Upper	14
#5	4	8	9.3 ± 1.4	2.2 ± 0.3	185 ± 20	Rt-Upper	20
#6	4	7	10.6 ± 2.5	2.8 ± 0.5	122 ± 14	Lt-Lower	13
#7	4	7	13.4 ± 1.8	3.0 ± 0.4	130 ± 25	Lt-Lower	14
#8	4	7	11.0 ± 3.4	3.4 ± 0.9	145 ± 10	Lt-Lower	16
#9	4	7	10.5 ± 4.3	3.3 ± 1.0	160 ± 17	Lt-Lower	18
#10	4	7	8.9 ± 2.2	3.4 ± 1.1	138 ± 12	Lt-Lower	15

#### Respiratory motion data in the respiratory-gating device

Fig. 1(b) shows the respiratory motion data assumed in the respiratory-gating device. The gating window was determined in the first few cycles even when the respiratory motion data change in terms of the amplitudes and periods of respiration in each cycle during the irradiation, as shown in Fig. 1(a). Irradiation was performed continuously in the gating window set assuming that the amplitude and period of the patients’ respiration are constant during irradiation, as shown in Fig. 1(b). This device was operated for irradiation despite any changes in the amplitude position of the target during irradiation, regardless of the baseline drift.

### Quantitative of the baseline drift

An index that considers not only the magnitude of amplitude position of the target but also the time (period) should be used to quantify the baseline drift. This is because the baseline drift is caused by two factors: the respiratory cycle and expiratory position of the target.

**Fig. 2:**
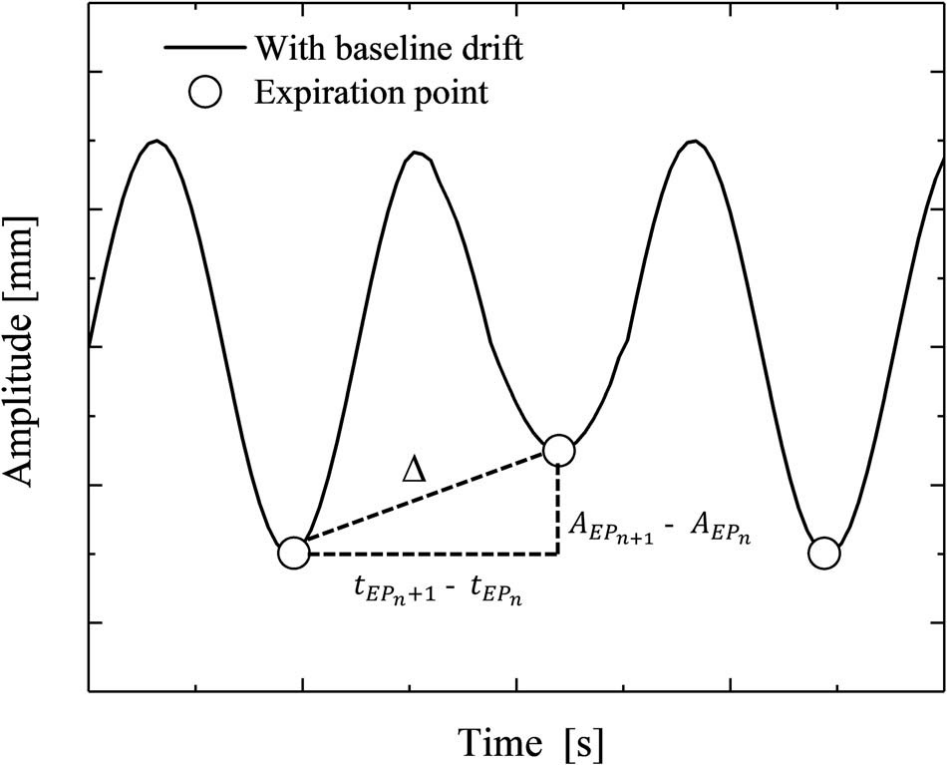
Schematic of the baseline drift. The solid line is the patient of the intra-beam respiratory motion data with baseline drift. The circles are expiration target positions in each cycle acquiring the in-house program. The $\Delta$ was defined as the inclination between the detected expiration point and the expiration point in the next cycle in AP, CC and LR directions.

The baseline drift $\Delta$ was calculated as shown in Fig. 2. The expiration points for each cycle were obtained based on the SciPy module, which contains a variety of toolboxes dedicated to the common problems in scientific computing in Python 3.6.6. The baseline drift $\Delta$ was defined as the inclination between the detected expiration point and expiration point in the next cycle in AP, CC and LR directions obtained using an in-house programme.

(1) \begin{equation*}\qquad\qquad \Delta \left[\mathrm{mm}/\mathrm{s}\right]=\frac{\left|{A}_{EP_{n+1}}-{A}_{EP_n}\right|}{t_{EP_{n+1}}-{t}_{EP_n}} \end{equation*}

where ${A}_{EP_n}$ and ${A}_{EP_{n+1}}$ are the expiration points of the amplitude in each cycle, and${t}_{EP_n}$ and ${t}_{EP_{n+1}}$ are times of the expiration points in each cycle.

### Calculation of residual motion

**Fig. 3:**
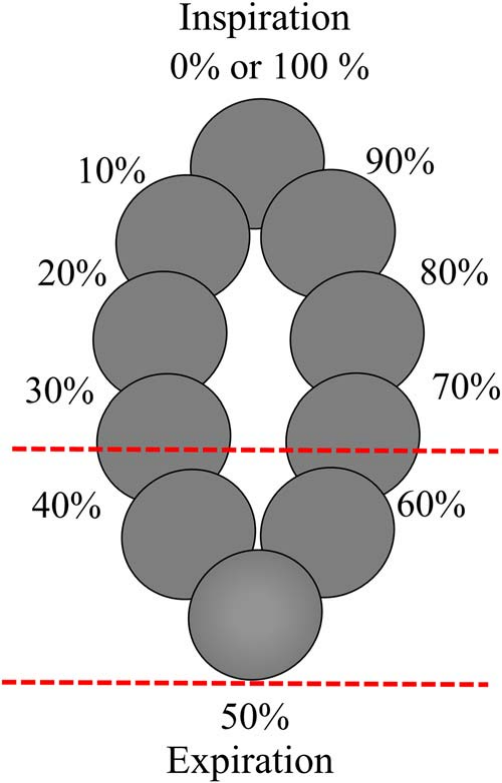
Schematic of the residual motion. The spheres were shown the position of 10th division of a cycle for the CTV. The region between the two red dotted lines indicates the gating window for irradiation in expiration phase. The residual motion was defined as the displacement of the target per cycle between the two red dotted lines.

The residual motions in the phase-gating and amplitude-gating lung SBRT were calculated using the in-house software. Fig. 3 shows an example of the residual motion in the expiration phase when the total respiratory wave is phase normalised for each cycle. The residual motion was defined as the displacement of the target per cycle within the gating window such as the expiration, transition and inspiration phases.

**Fig. 4:**
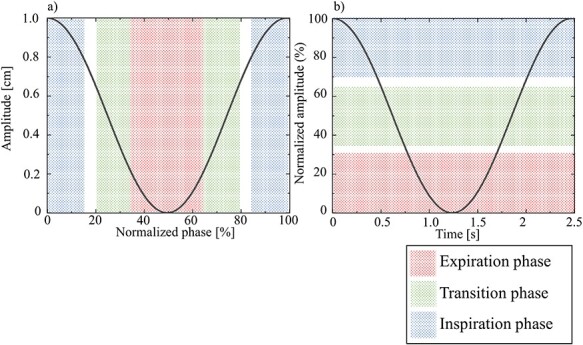
The gating windows of the phase- and amplitude-gating method. (a) In the phase-gating method, the respiratory wave is normalised as one respiratory cycle with the expiration phase at 35–65% (red region), transition phase at 20–35%, 65–80% (green region), inspiration phase at 0–15% and 85–100% (blue region) set as the gating parameters. (b) In the amplitude-gating method, the respiratory waves were normalised to the minimum and maximum amplitudes of the first three cycles in the respiratory waves with the gating window set for the expiration phase (0–30%; red region), transition phase (35–65%; green region) and inspiration phase (70–100%; blue region).

The method of determining the gating window was simulated as per the method used for the respiratory-gating system. For phase-gating lung SBRT, the respiratory wave was normalised for one respiratory cycle as follows: expiration phase, 35–65%; transition phase, 20–35%, with 65–80%; inspiration phase, 0–15%, with 85–100% set as the gating parameter (Fig. 4a). Amplitude-gating lung SBRT was normalised to the minimum and maximum amplitudes of the first three cycles of the respiratory waves, with a gating window set for the expiration phase (0–30%), transition phase (35–65%) and inspiration phase (70–100%; Fig. 4b). The residual motion is defined as the respiratory motion data within these gating window settings.

### Calculation of dose distribution

#### Calculation of the probability

The dose distribution was calculated using the in-house software using the method reported by van Herk *et al.* [17]. The CTV motion was assumed to be the motion of each respiratory motion data point.

**Fig. 5:**
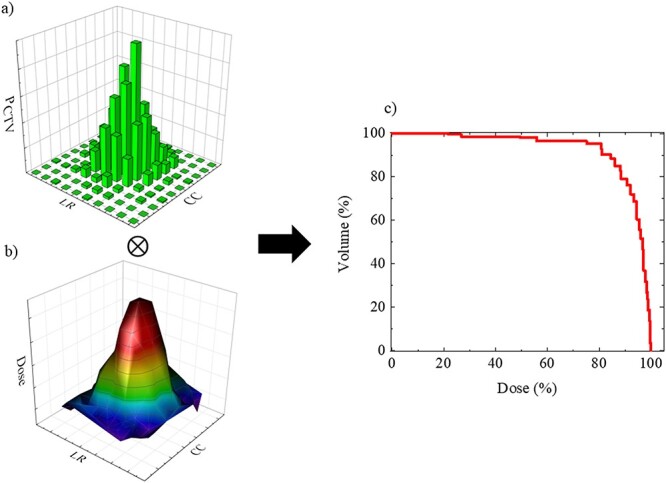
**(**a) This figure is an example of the probability distribution calculated as per the CTV position during the delivery of the dose to target in the in LR and CC directions. (b) This figure is an example of the reference dose distribution for simulating the respiratory gating lung SBRT was calculated by the TPS in LR and CC directions. The probability and the dose distribution were actually calculated in LR, CC and AP directions. (c) The DVH in CTV was convoluted the probability and the reference dose distribution in LR, CC and AP directions. $\otimes$ is the convoluted operator.

The probability with the CTV was calculated by considering each detail of the CTV to clarify the effect of the baseline drift in the phase- and amplitude-gating lung SBRT. The CTV that included these respiratory motion data were extracted as the probability data (Fig. 5a). Fig. 5a shows the probability distribution calculated as per the CTV position during the delivery of the dose to target in the AP, CC and LR directions. The probability in the CTV was defined as:

(2) \begin{equation*} {P}_{\mathrm{CTV}}={\int}_{A_{\mathrm{lower}}}^{A_{\mathrm{upper}}}Q(z) dz \end{equation*}

where $Q(z)$ is the shape of the probability density function. ${A}_{\mathrm{upper}}$ and ${A}_{\mathrm{lower}}$ are the maximum and minimum amplitudes in the gating window, respectively. ${P}_{\mathrm{CTV}}$ values were calculated for each of the expiration, transition and inspiration phases in the phase- and amplitude-gating methods.

#### Calculation of reference dose distribution

The reference dose distribution was calculated in the AP, CC and LR directions using the treatment planning system (TPS) for simulating the respiratory-gating lung SBRT. Table 2 lists the calculation conditions of the reference dose distribution used to calculate the Dose volume histogram (DVH) in the phase- and amplitude-gating lung SBRT. The TPS was RayStation ver. 6.2.2 (RaySearch Lab., Stockholm, Sweden), which defined the planning target volume (PTV) assuming CTV + 5 mm within the phantom (CIRS Inc., Norfolk, VA, USA) CT images for simulating the respiratory-gating lung SBRT [6, 9, 10, 18]. Further, 6 MV X-rays were used. Five beam non-coplanar 3D-CRT was applied. The prescribed dose was set at 48 Gy/4 Fr. The diameter of the CTV was set at 23 mm considering the average CTV of the included patients. The reference dose distribution was calculated using five beams for simulating the lung SBRT. The PTV was covered with 80% isodose.

**Table 2:** The conditions of the reference dose distribution. Reference dose distribution was calculated in the AP, CC and LR directions using the TPS for simulating respiratory gating lung SBRT. This dose distribution was used calculating these DVH for phase- and amplitude-gating SBRT

TPS	RayStation ver 6.2.2 (Raysearch Lab., Stockholm, Sweden)
Phantom	Thorax phantom (CIRS Inc., Norfolk, VA, USA)
Energy	6 MV-X
Number of beams	5 non-coplanar
CTV	23-mm sphere
PTV	CTV + 5 mm
Gy/Fr	48 Gy/4 Fr

#### Calculation of dose distribution and DVH in CTV

Because sorting by the amplitude-gating method was difficult using the current CT system, we adopted the method used by van Herk *et al.* [17]. This method allows us to consider the phase and amplitude in identical ways. The dose distribution and DVH (Fig. 5c) convoluted the probability (Fig. 5a) and the reference dose distribution (Fig. 5b) in the AP, CC and LR directions. The interplay effect did not occur in this study because the 3D-CRT irradiation method was used. The numerical value of the homogeneity index (HI) as defined in the International Commission on Radiation Units and Measurements (ICRU) report 83 was used to analyse the DVH in the phase- and amplitude-gating lung SBRT [19]. The HI is the index of dose uniformity for the target, and it is defined as:

(3) \begin{equation*}\qquad \qquad\qquad HI=\frac{\left({D}_2-{D}_{98}\right)}{D_{50}} \end{equation*}

where ${D}_2$ refers to the dose delivered to 2% volume of the CTV; ${D}_{98}$ refers to the dose delivered to 98% volume of the CTV; and ${D}_{50}$ refers to the dose delivered to 50% volume of the CTV. The closer HI is to 0, the better is the dose uniformity for the CTV.

## RESULTS

### Quantitative study of the baseline drift

**Fig. 6:**
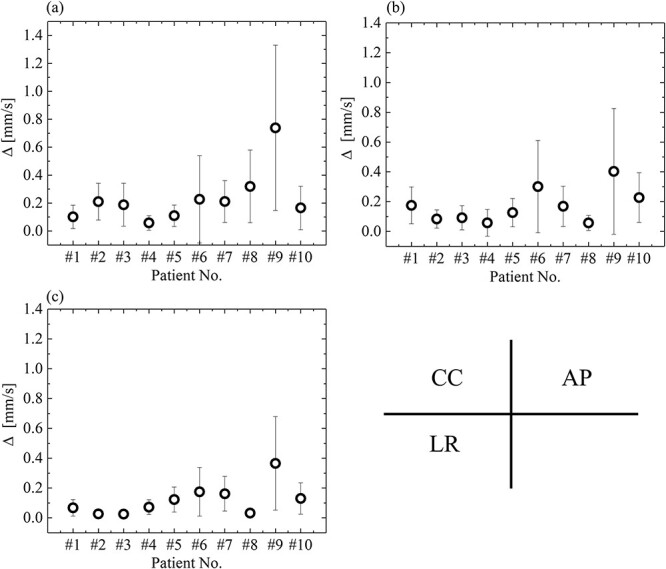
The baseline drift was quantified as the $\varDelta$ in AP, CC and LR directions. The baseline drift $\varDelta$ was calculated by equation (1). The vertical axis represents the $\varDelta$ [mm/s]. The horizontal axis represents the Patient No. of these intra-beam respiratory motion data.

The baseline drift $\Delta$ during the irradiation was quantified using equation (1). Fig. 6 shows the baseline drift values of the intra-beam respiratory motion data for 10 patients in the AP, CC and LR directions. The baseline drift $\Delta$ was the largest in the CC direction. The value was distributed from 0.06 to 0.40, 0.06 to 0.74 and 0.03 to 0.37 mm/s in the AP, CC and LR directions, respectively, for the intra-beam respiratory motion data of the 10 patients. In patient No. 9, where the target was in the lower lobe, the baseline drift $\Delta$values were 0.40, 0.74 and 0.37 mm/s in the AP, CC and LR directions, respectively, and the magnitude of the baseline drift was the largest among the 10 patients.

Patients No. 4 and No. 5, in which the targets were in the upper lobes, had baseline drifts $\Delta$ less than 0.13 in all directions and had the smallest magnitudes of baseline drift among the 10 patients.

### Analysis of residual motion

**Fig. 7:**
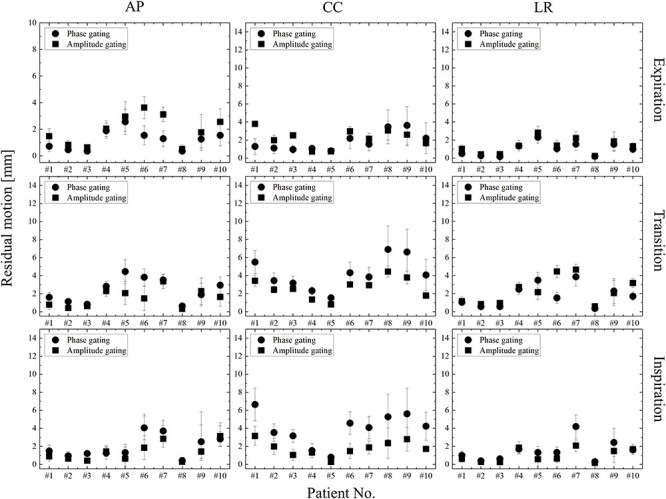
Residual motions of the phase- and amplitude-gating methods in the AP, CC and LR directions. In the phase- and amplitude-gating methods, (the upper side) for the expiration phase: the gating window set at 35–65% and 0–30%; (the middle side) the transition phase: the gating window is set at 20–35% and 65–80%; and (the lower side) the inspiration phase: the gating window is set at 0–15% and 85–100% and 70–100%, respectively.

The residual motion was quantified from the intra-beam respiratory motion data in lung cancer patients. The residual motion for the expiration, transition and inspiration phases are shown in Fig. 7. Because of baseline drift, the residual motion in each respiratory phase was different for each patient. In the expiratory phase, the phase-gating method showed that the residual motion was up to 2.5 mm smaller. In contrast, in the transition and inspiration phases, the amplitude-gating method reduced the residual motion by up to 3 mm. For patient No. 9 with the target in the lower lobe, the difference between the phase- and amplitude-gating of the 3D residual motion was −0.48, −2.37 and − 3.14 mm in the expiration, transition and inspiration phases, respectively. The differences among the 3D residual motions of the different gating methods was within 1.0 mm in the expiratory phase but more than 2 mm in the transition and inspiration phases. The difference for patient No. 4, with the target in the upper lobe, between the phase- and amplitude-gating of the 3D residual motion was 0.02, −0.59 and 0.08 mm in the expiratory, transition and inspiration phases, respectively. The difference for patient No. 5, with the target in the upper lobe, between the phase- and amplitude-gating methods of the 3D residual motion was 0.61, −2.77 and − 1.11 mm for the expiration, transition and inspiration phases, respectively. For patient No. 4, the differences in the 3-dimensional residual motion between the different gating methods was within 1.0 mm for all phases.

### Analysis of dose distribution

**Fig. 8:**
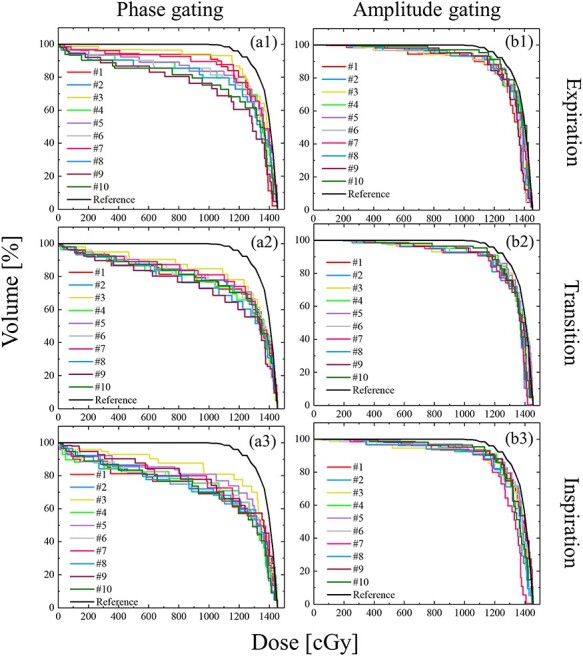
DVH in the phase- and amplitude-gating SBRT calculated on the basis of the probability of the CTV in the intra-beam respiratory motion of the patients. The vertical axis represents the absolute dose in the CTV. The horizontal axis represents the relative volume of the CTV. Fig. a1–a3 shows the DVH of the phase gating method with the baseline drift in the expiration, transition and inspiration phases. Fig. b1–b3 shows the DVH of the phase gating method with baseline drift in the expiration, transition and inspiration phases. The DVH of the ‘Reference’ was calculated as specified in section 2.4.2.

The DVH was calculated on the basis of the probability of the target in the intra-beam respiratory motion of the patients. The DVH was calculated using the convolution of the probability. The reference dose distribution in the phase- and amplitude-gating lung SBRT are shown in Fig. 8. For reference, the DVH of the reference dose distribution was calculated as specified in section 2.4.2.

Table 3 lists the HI for all gating methods and phases. To evaluate the dose uniformity to the target, HI was calculated by using equation (3). The HI of the phase-gating method increased by up to 0.63, 0.51 and 0.54 in the expiration, transition and inspiration phases compared to the HI of the amplitude-gating method. The HI of the phase-gating method tended to increase because the target was irradiated despite changes in the amplitude position of the target depending on the period.

**Table 3:** HI of the phase- and amplitude-gating lung SBRT, compared the reference dose distribution. The numerical value to analyse the DVH in phase- and amplitude-gating lung SBRT used HI as defined by equation ( 3)

		Patient No.									
		#1	#2	#3	#4	#5	#6	#7	#8	#9	#10
Reference dose distribution		0.22									
Phase gating	Ex	1.03	0.95	0.95	1.05	1.04	1.07	1.03	1.09	1.11	1.08
	Tra	1.07	1.05	1.02	1.07	1.05	1.04	1.08	1.09	1.07	1.05
	In	1.08	1.12	1.02	1.09	1.10	1.08	1.08	1.09	1.01	1.11
Amplitude gating	Ex	0.84	0.82	0.83	0.71	0.68	0.79	0.74	0.72	0.71	0.45
	Tra	0.67	0.71	0.71	0.65	0.64	0.69	0.57	0.72	0.65	0.58
	In	0.67	0.68	0.91	0.64	0.63	0.85	0.85	0.82	0.70	0.57

## DISCUSSION

The baseline drift during the irradiation was quantified using the intra-beam respiratory motion data in the LR, CC and AP directions. The drifts were calculated on the basis of the inclination between the maximum expiration point and the value less than or more than the maximum expiration point obtained using the in-house programme. The baseline drift $\Delta$ was distributed from 0.06 to 0.40, 0.06 to 0.74 and 0.03 to 0.37 mm/s in the AP, CC and LR directions, respectively, for the intra-beam respiratory motion data of the 10 patients. The baseline drift was greater in the CC direction than in the other directions. Takao *et al.* reported that the magnitude of the baseline drift increased with the treatment time [7]. However, the magnitude of the baseline drift may be small in this study because the intra-beam respiratory motion was evaluated in this study. In previous studies, the baseline drift could not be specified for analysis at ≥1 min intervals regardless of the rapid changes in the expiration target position [5, 7]. In this study, the baseline drift during the irradiation was quantified by analysing the expiration target position in every respiratory cycle, considering the abrupt changes in the expiratory target position. Although the baseline drift was obtained for each beam, it was faithfully reproduced from the intra-beam respiratory motion to provide clinically useful data. Patients No. 4 and No. 5, with targets in the upper lobes, had a Δ value less than 0.13, suggesting a smaller baseline drift than that observed in the data for targets located in the lower lobe. Takao *et al.* also reported similar results. It is necessary to consider the baseline drift in the respiratory-gating lung SBRT for patients whose targets are located in the lower lobe [7].

Our study shows that the maximum differences in the residual motion between the phase- and amplitude-gating methods in the CC direction were 2.1, 1.5 and − 0.4 mm for the inspiration, transition and expiration phases, respectively. In the expiration phase, the phase- and amplitude-gating methods did not show any major differences. Berbeco *et al.* showed an approximately 30% reduction in the residual motion in three of the irregularly respiring patients using the amplitude-gating method as compared with the phase-gating method in the expiration phase [13]. However, this study shows that the residual motion did not undergo a major change in the expiration phase. In the amplitude-gating method, the residual motion in the inspiration and transition phases decreased by 2.1 and 1.5 mm, respectively, compared with that in the phase-gating method. The residual motion did not depend on the baseline drift as the results for the respiration phases of residual motion were uneven regardless of the baseline drift.

We evaluated the residual motion of the fiducial markers assuming that it corresponds to the target position. The geometric relationship between the target and fiducial marker placed in the same lung lobe may change slightly during respiration. The respiratory-gating device performed gating irradiation assuming that the body surface signals and target position were coincident. Berbeco *et al.* reported that external markers placed on the body surface correlate well with the movement of the fiducial markers [13, 14]. However, baseline drift during irradiation can occur regardless of phase and amplitude-gating method. Note that the respiratory-gating device may overlook any possible baseline drift within the gating window.

The dose distributions in the two different respiratory-gating methods were calculated using the intra-beam respiratory motion data with the baseline drift. The calculated DVH was based on the probability of the delivered dose at the CTV. The HI of the phase-gating method increased by a maximum of 0.63, 0.51 and 0.54 in the expiration, transition and inspiration phases, respectively, compared to the HI of the phase-gating method. The respiratory-gating lung SBRT was performed by setting the gating window in the first few cycles assuming constant cycle and amplitude of respiration during irradiation, as shown in Fig. 2. Hence, irradiation was performed in the phase-gating method despite any changes in the amplitude position of the target in each cycle. In contrast, the amplitude-gating method enables irradiation considering the amplitude position of the target because the gating window is set for the amplitude position of the target motion.

The amplitude-gating method can be adopted for irradiation in lung SBRT, and the lung may be irradiated in the inspiratory phase to increase the lung volume and reduce the dose to the normal lung tissue [14, 20]. This study suggests that the amplitude-gating method was effective for irradiation in the inspiratory phase because the DVH curve in the inspiratory phase was closer to that of the reference dose distribution in the amplitude-gating method than in the phase-gating method. The effectiveness of the amplitude-gating method has been reported previously, and this method may be applied to irregular respiratory motion data, such as respiratory motion with a baseline drift [10, 11].

## CONCLUSION

This study aimed to quantitatively clarify the baseline drift for each respiratory cycle in two respiratory-gating methods using the intra-beam respiratory motion data of lung cancer patients. The baseline drift was quantified by analysing the expiration target position in every respiratory cycle during the intra-beam respiratory motion. In the phase-gating method, irradiation is performed even if the amplitude position of the target changes in each cycle. In contrast, in the amplitude-gating method, the gating window is set based on the amplitude position of the target, thus enabling irradiation considering the amplitude position of the target. The respiratory-gating methods and the respiratory phase in the respiratory-gating lung SBRT with the baseline drift must be determined based on the respiratory motion of patients.
